# An Update on the Current State of Management and Clinical Trials for IgA Nephropathy

**DOI:** 10.3390/jcm10112493

**Published:** 2021-06-04

**Authors:** Chee Kay Cheung, Arun Rajasekaran, Jonathan Barratt, Dana V. Rizk

**Affiliations:** 1 Department of Cardiovascular Sciences, University of Leicester, Leicester LE1 7RH, UK; ckc15@le.ac.uk (C.K.C.); jb81@le.ac.uk (J.B.); 2 John Walls Renal Unit, University Hospitals of Leicester NHS Trust, Leicester LE5 4PW, UK; 3 Division of Nephrology, Department of Medicine, University of Alabama at Birmingham, ZRB 614, 1720 2nd Avenue South, Birmingham, AL 35294, USA; arajasekaran@uabmc.edu

**Keywords:** IgA, IgA nephropathy, clinical trials

## Abstract

IgA nephropathy remains the most common primary glomerular disease worldwide. It affects children and adults of all ages, and is a leading cause of end-stage kidney disease, making it a considerable public health issue in many countries. Despite being initially described over 50 years ago, there are still no disease specific treatments, with current management for most patients being focused on lifestyle measures and renin-angiotensin-aldosterone system blockade. However, significant advances in the understanding of its pathogenesis have been made particularly over the past decade, leading to great interest in developing new therapeutic strategies, and a significant rise in the number of interventional clinical trials being performed. In this review, we will summarise the current state of management of IgAN, and then describe major areas of interest where new therapies are at their most advanced stages of development, that include the gut mucosal immune system, B cell signalling, the complement system and non-immune modulators. Finally, we describe clinical trials that are taking place in each area and explore future directions for translational research.

## 1. Introduction

IgA nephropathy (IgAN) remains the most common primary glomerular disease in the world. It often affects younger adults, and in approximately 30% of patients it progresses to end-stage kidney disease (ESKD) within 20 years of diagnosis, placing a considerable burden on individuals, carers and healthcare systems globally. To date there is no approved disease-specific treatment available. The role of corticosteroids in the management of IgAN is uncertain, and there has been no consistent evidence to support the use of other existing immunosuppressive agents. Over the past few decades, significant advances have been made in understanding the complex pathogenesis that underlies IgAN. This has driven an explosion of interest in developing new therapeutic strategies for this condition, and several global phase II and phase III clinical trials are currently underway. In this review, we will focus on current treatment guidelines and new therapies for IgAN that range from being in the initial stages of development to the late phases of clinical trials.

## 2. Current Treatment Strategies

Despite advances in the understanding of the underlying pathogenic mechanisms in IgAN, there are currently no approved treatments that can specifically alter the production of galactose-deficient IgA1 (Gd-IgA1) nor its corresponding autoantibody that are central to the disease process, IgA1-immune complex formation, or their deposition within the glomerular mesangium.

Long-term registry data have shown that patients with IgAN who have preserved kidney function, non-visible haematuria and minimal proteinuria <0.5 g/day are at low risk of progressive kidney disease, and do not require disease-specific treatment [1]. However, these patients should be followed up at least annually, so that any worsening of proteinuria, development of chronic kidney disease (CKD) or hypertension can be detected and managed appropriately.

The primary focus of management for those who have proteinuria above this threshold should be on optimized goal-directed supportive care. This includes renin-angiotensin-aldosterone system (RAAS) blockade, with either an angiotensin-converting enzyme inhibitor (ACEi) or angiotensin II receptor blocker (ARB), but not both, to the maximum tolerated amount, and addressing overall cardiovascular risk, including strict blood pressure control (to a recommended target of <130/80 mm Hg for those with proteinuria > 0.3 g/day and <125/75 mm Hg when proteinuria is >1 g/day) [2], dietary sodium reduction, smoking cessation if appropriate, weight control, and exercise. The Supportive versus immunosuppressive Therapy for the treatment of Progressive IgA Nephropathy (STOP-IgAN) trial has provided robust evidence to support this approach [3]. All participants in this trial underwent a 6-month run-in period, where an intensive program of supportive care was provided utilizing the measures outlined above. A key finding was that around one-third of participants, originally thought by their treating nephrologist to be suitable for treatment of their disease with immunosuppression, were no longer eligible to continue in the trial after this period, as proteinuria had fallen to below the set criteria to proceed.

A substantial proportion of patients will have persistent proteinuria of >1 g/day despite these measures, and observational data have demonstrated that these patients are at high risk of progressive kidney disease and ESKD. Reducing proteinuria to below this threshold is also associated with improved renal outcomes [4]. Long-term follow-up data from the STOP-IgAN trial have shown that IgAN still has a poor prognosis in those with persistent proteinuria, even with optimized supportive care, with almost half of the participants reaching the composite of death, ESKD, or decline in estimated glomerular filtration rate (eGFR) of over 40% over a median time interval of 7.4 years [5]. In the next sections, we will discuss potential additional treatments to supportive care.

## 3. Systemic Corticosteroid Treatment

Corticosteroids have long been used in the management of IgAN, due to their anti-inflammatory and immunosuppressive effects, but their role is controversial. Many of the clinical trials that have supported their use were conducted at a time when the concept of optimized supportive care was not well established, meaning that trial participants were not consistently treated to strict blood pressure targets and the use of RAAS inhibitors was variable. In addition, data regarding adverse events were often not systematically collected.

In an early randomized controlled trial by Pozzi et al., treatment with corticosteroids resulted in significantly reduced proteinuria and prevented progression to ESKD over a 10-year follow-up period [6]. The treatment regimen included pulsed IV methylprednisolone at induction, and at months 2 and 4, with alternate day oral prednisolone for 6 months. This would be expected to be associated with significant toxicity, yet only one significant adverse event of steroid-induced diabetes mellitus was recorded. In keeping with standard of care at the time, RAAS blockade use was low and the achieved blood pressure was higher than current treatment targets. Subsequent studies by Manno et al. and Lv et al. also reported a beneficial effect from a 6-month course of corticosteroids in high-risk patients, defined by having proteinuria > 1 g/day [7,8]. However, temporary discontinuation of RAAS blockade was mandated before re-introduction at baseline in both trials, and it is possible that a number of included patients would have responded to optimized RAAS blockade and supportive care alone.

Two recent RCTs have addressed these points, the STOP-IgAN and the Therapeutic Evaluation of Steroids in IgA Nephropathy Global (TESTING) studies, by including a run-in period where supportive care could be optimized. STOP-IgAN, conducted in Germany, compared intensive supportive care to immunosuppressive therapy (corticosteroids if estimated glomerular filtration rate (eGFR) was ≥60 mL/min/1.73m^2^, or cyclophosphamide followed by azathioprine with prednisolone if eGFR was between 30–59 mL/min/1.73m^2^) in patients at high risk of progressive kidney disease [3]. Although a reduction in proteinuria was observed in the immunosuppressed group compared to supportive care alone, there was no difference in eGFR decline between the groups, and more episodes of infection (with one fatal pneumonia) and other adverse events, including malignancy, impaired glucose tolerance and weight gain, occurred in those receiving immunosuppression. In a follow-up study, no significant difference in renal outcomes was observed between the immunosuppressed and supportive care groups at a median of 7.4 years follow-up [5]. The TESTING trial, conducted mainly in centers in China, of oral methylprednisolone vs. placebo in IgAN patients at high risk of progression was stopped early due to an excess of serious adverse events in the methylprednisolone group, including one fatal case of *Pneumocystis jirovecii*, although interestingly, there was a significant reduction in those reaching the composite renal outcome (40% reduction in eGFR, ESKD, or death due to renal failure) in this group [9]. Important differences between these two RCTs have been discussed in detail previously [10].

Currently, the risk-benefit ratio for corticosteroids in the treatment of IgAN remains uncertain, and significant questions remain regarding their optimum dose and duration, and patient selection. The TESTING low-dose study will assess whether a lower dose of methylprednisolone together with *Pneumocystis jirovecii* prophylaxis can be beneficial while avoiding the rate of serious adverse events observed in the earlier trial, and this is due to be reported in 2023 (ClinicalTrials.gov Identifier: NCT01560052). It should also be noted that corticosteroids are typically pursued as part of treatment in the rare circumstances where IgAN is associated with nephrotic syndrome, or with rapidly progressive glomerulonephritis. Both scenarios have been excluded from clinical trials addressing the benefit of steroids in the treatment of IgAN.

Forthcoming Kidney Disease: Improving Global Outcomes (KDIGO) guidelines emphasize that although patients with IgAN who have proteinuria >1 g/day despite 90 days of optimized supportive care can be considered for corticosteroid therapy, their clinical benefit is not established, and that it is much preferred that such patients be offered an opportunity to take part in a therapeutic clinical trial.

## 4. Clinical Trial Design in IgAN

There has been a welcomed increase in the number of clinical trials being performed in IgAN over the past decade. However, a number of difficulties are inherent to studying this disease. Firstly, it should be recognized that IgAN may not be a single disease, but instead may represent a common histological endpoint towards which distinct pathogenic mechanisms may contribute [11]. Its clinical presentation and rate of progression is highly variable between individuals, with evidence that these factors vary according to geographical location and ethnicity. The implication of this is that findings from trials conducted in certain populations may not be applicable to others. Secondly, in most cases, IgAN is a slowly progressive disease, where the traditional renal endpoints of death, dialysis, or doubling of serum creatinine may take many years to occur. This has previously meant that clinical trials have been prohibitively expensive and difficult to conduct, especially as IgAN is a rare disease. Incorporation of a ‘pre-‘ and ‘post-treatment’ kidney biopsy in clinical trials can yield significant mechanistic insights into a certain drug’s effectiveness, although this is an invasive procedure that is associated with a small risk of complications, and would not be accepted by all participants. Recent data have demonstrated that proteinuria reduction and the rate of change/slope of eGFR decline are accurate surrogate endpoints for these renal outcomes [12,13]. Trial-level analysis of 13 controlled trials in IgAN by a Kidney Health Initiative workgroup demonstrated an association between proteinuria reduction and effects on a composite of time to doubling of serum creatinine, ESKD or death, that was independent of the therapeutic intervention used [13]. These endpoints have recently been approved by the US Food and Drug Administration (FDA) for use in clinical trials in IgAN, generating further interest in drug development in this field.

In the following sections, we will describe the systems in IgAN that are affected, with a view to discussing interventional treatment strategies targeting these areas.

## 5. The Gut Mucosal Immune system and IgAN

**Figure 1:**
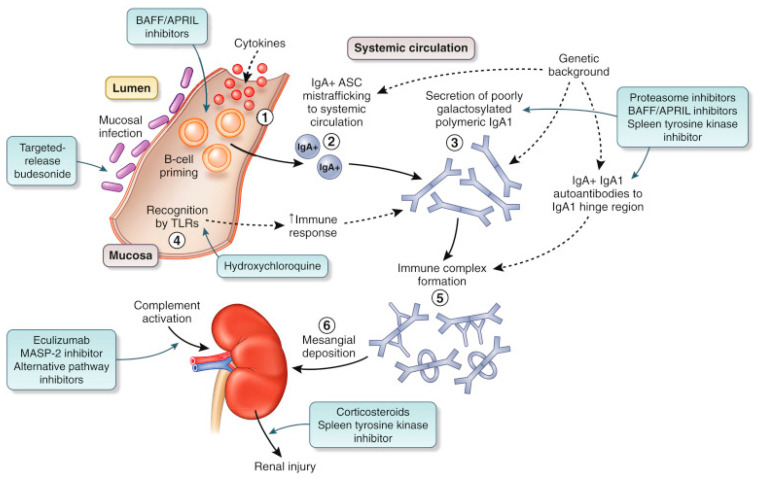
Proposed pathogenesis of IgAN and novel treatment strategies. (**1**) Mucosal infection primes naïve B cells to class switch to become IgA^+^ antibody secreting cells (ASCs) through T cell-dependent (cytokine mediated) and T cell-independent (Toll-like receptor (TLR) ligation) pathways. (**2**) Some IgA^+^ ASC mis-home to the systemic compartment during lymphocyte trafficking. (**3**) Displaced IgA^+^ ASCs take up residence in systemic sites and secrete normal ‘mucosal-type’ poorly galactosylated (galactose deficient) and polymeric IgA1 into the systemic circulation. (**4**) IgA1 secretion by displaced mucosal ASC is augmented by TLR ligation from mucosal-derived pathogen-associated molecular patterns, which have entered the systemic compartment. (**5**) IgA1 immune complexes form in the systemic circulation. Poorly galactosylated polymeric IgA1 molecules are the substrate for immune complex formation and combine with IgG and IgA autoantibodies reactive to exposed neoepitopes in the poorly galactosylated IgA1 hinge region. (**6**) IgA1 immune complexes deposit in the mesangium through a combination of mesangial trapping and increased affinity of poorly galactosylated IgA1 for extracellular matrix components. Immune complex deposition triggers a series of downstream pathways leading to glomerular injury and tubulointerstitial scarring. APRIL, a proliferation-inducing ligand; BAFF, B cell activating factor; MASP-2, mannan-binding lectin-associated serine protease-2. Reprinted from ref. [10], with permission from Elsevier.

There is an increasing recognition of the role of the gut-associated lymphoid tissue (GALT), particularly the Peyer’s patches, in the generation of the pathogenic Gd-IgA1 molecules [14,15,16,17]. Gd-IgA1 enters the systemic circulation either via direct passage and/or displacement of GALT-derived B cells to systemic sites, including the bone marrow, secondary to an error in the homing mechanism, and eventually leads to secretion of mucosal-type Gd-IgA1 into the bloodstream (Figure 1) [18]. A novel, oral, targeted-release formulation (TRF) of the glucocorticoid, budesonide (NEFECON^®^) was designed to deliver the drug to the distal ileum where the highest concentration of mucosal Peyer’s patches reside to reduce Gd-IgA1 release into the circulation [19,20,21] (Table 1). An exploratory phase IIa trial of TRF-budesonide in 16 IgAN patients revealed a statistically significant reduction in proteinuria and was also well tolerated [22]. Subsequently, the Targeted-Release Budesonide Versus Placebo in Patients with IgAN (NEFIGAN) trial compared TRF-budesonide (*n* = 100) with placebo (*n* = 50) in a phase IIb randomized, controlled, double-blind clinical trial [21]. Enrolled IgAN patients had persistent proteinuria, defined by a urine protein-to-creatinine ratio (UPCR) > 0.5 g/g or proteinuria or at least 0.75 g/day, despite optimal RAAS blockade. The study consisted of a 6-month run-in, 9-month treatment phase, and 3 months of follow-up. Two doses of TRF-budesonide (16 mg/day; 8 mg/day) were compared to placebo. The baseline proteinuria was 1.2 g/day (interquartile range: 0.9–2 g/day), and the baseline eGFR was 78 ± 5 mL/min/1.73m^2^. The study achieved its primary end point of mean change in proteinuria with a UPCR reduction by an average of 24.4% (−0.212 g/g) in the combined treatment arms versus an increase by 2.7% (+ 0.024 g/g) in the placebo arm (geometric least-square mean change in UPCR vs. placebo 0.74; 95% CI 0.59-0.94; *p* = 0.006). Importantly, this effect was sustained throughout the follow-up period of the investigational product. The secondary endpoint of change in eGFR was also achieved due to a decrease by 9.8% (*p* = 0.001) in the placebo group over the study timeframe which was somewhat surprising. No serious adverse events, including infections, were reported in the treatment group [21]. The safety profile of TRF-budesonide is proposed to be superior to high dose systemic corticosteroid therapy given its extensive first pass metabolism with <10% of budesonide entering the systemic circulation [23].

The promising results of the NEFIGAN trial [21] led to the design of the ongoing phase III Efficacy and Safety of TRF-budesonide in Patients With Primary IgA Nephropathy (NefIgArd) study (ClinicalTrials.gov Identifier: NCT03643965). This multi-national, randomized, double-blind, placebo-controlled, phase III clinical trial aims to evaluate the efficacy, safety, and tolerability of TRF-budesonide 16 mg/day formulation in the management of patients with biopsy-proven primary IgAN at risk of progressing to end-stage kidney disease (ESKD), despite maximum tolerated RAAS blockade. Part A of the trial encompasses a 9-month treatment and a 3-month follow-up period. Part B comprises a 12-month observation period. The primary goal of Part A is to assess the effect of TRF-budesonide 16 mg/day on 24-h UPCR values over 9 months when compared to placebo. The primary objective of Part B is to assess the effect of the investigational drug on kidney function. The study aims to recruit 360 IgAN patients across 20 countries and is projected to have achieved its recruitment goal in March 2021. Interim results of the NefIgArd trial have confirmed the earlier phase IIb findings of the NEFIGAN trial [24]. The primary endpoint analysis, that included 199 patients with primary IgAN, showed a 31% mean reduction in 24-h UPCR in the TRF-budesonide 16 mg/day arm versus baseline, with placebo showing a 5% mean reduction versus baseline, resulting in a 27% mean reduction in 24-h UPCR at 9 months (*p* = 0.0005) of the TRF-budesonide 16 mg/day arm versus placebo. The secondary endpoint data on eGFR showed a treatment benefit of 7% versus placebo at 9 months, reflecting stabilization in the treatment arm and a 7% decline in eGFR in the placebo arm (*p* = 0.0029). This reflected an absolute decline in eGFR of 4.04 mL/min/1.73m^2^ in the placebo group over 9 months compared to a 0.17 mL/min/1.73m^2^ eGFR decline in the treatment group. TRF-budesonide was generally well-tolerated [24].

## 6. Targeting B Cells

There is clear evidence that IgAN is an autoimmune disease. Production of Gd-IgA1 and glycan-specific IgG and IgA autoantibodies against Gd-IgA1 lead to the formation of IgA-containing immune complexes that deposit within the mesangium, causing subsequent effects on mesangial cells, podocytes and proximal tubular cells that drive glomerular and tubulointerstitial inflammation and fibrosis. Glycan-specific IgG autoantibodies found at increased levels in IgAN correlate with the prognosis and can be detected within mesangial deposits [25,26,27]. Targeting B cells in IgAN is therefore an attractive therapeutic strategy.

Effective B cell maturation and survival is dependent on BAFF (B cell activating factor) and APRIL (a proliferation inducing ligand). BAFF and APRIL bind to the tumor necrosis factor (TNF) superfamily receptors, BCMA (B cell maturation antigen), TACI (Transmembrane activator and calcium-modulating cyclophilin ligand interactor) and BAFF-R (BAFF-receptor).

There are several lines of evidence that this system plays an important role in IgAN. Transgenic mice that overexpress BAFF develop a hyper-IgA syndrome, and an IgAN-like renal phenotype [28,29]. Interestingly, this was dependent on the presence of gut commensal bacteria, presumably reflecting mucosal B cell activation. In vitro, tonsillar mononuclear cells, part of the mucosa-associated lymphoid tissue of Waldeyer’s ring, from patients with IgAN exposed to the bacterial motif CpG-oligodeoxynucleotides produced increased levels of BAFF and IgA [30]. This production was blocked by the inhibition of BAFF. Levels of serum BAFF are increased in IgAN, and correlate with disease severity, as measured by renal histology (increased mesangial hypercellularity, segmental glomerulosclerosis, and tubular atrophy/interstitial fibrosis) and serum creatinine at the time of renal biopsy [31]. APRIL also plays an important role in B cell maturation and survival, and is involved in the generation of IgA-secreting plasma cells. Tonsillar APRIL and Toll-like receptor (TLR) 9 levels have been shown to be increased in IgAN, and TLR-9 stimulation increased APRIL expression by tonsillar B cells [32]. In a study of patients with IgAN, serum levels of APRIL were associated with increased Gd-IgA1 and worse clinical presentation [33]. Genome-wide associated studies have identified *TNFSF13* as a risk allele for IgAN, and this encodes for APRIL [34]. This risk variant was also associated with higher serum levels of IgA in patients with IgAN. In a recent study, APRIL inhibition reduced serum IgA and immune complex levels, and reduced glomerular IgA deposition in the ddY mouse model of IgAN [35].

There has consequently been great interest in targeting these pathways in IgAN. Blisibimod is a selective BAFF inhibitor and has been tested in a phase II trial in IgAN. Interim analysis of results suggests that subcutaneous blisibimod may reduce proteinuria in IgAN and the full results of this trial are awaited (ClinicalTrials.gov Identifier: NCT02062684). Atacicept is a fully humanized fusion protein that contains the extracellular portion of TACI and inhibits both BAFF and APRIL signaling. Preliminary results from a Phase II trial suggest a dose-dependent effect on proteinuria levels [36]. This trial was terminated early due to slow recruitment, and further studies of atacicept in IgAN are planned. Studies of other inhibitors of BAFF and APRIL in IgAN are currently ongoing, including of VIS649, a humanised IgG2 monoclonal antibody that binds to and inhibits APRIL (ClinicalTrials.gov Identifier: NCT04287985), and BION1301, a humanized IgG4 monoclonal antibody, which is also directed against APRIL (ClinicalTrials.gov Identifier: NCT03945318).

Rituximab is a chimeric murine/human monoclonal antibody that targets the CD20 antigen expressed on the surface of pre-B and mature B lymphocytes, hence leading to B cell lysis upon binding. In contrast to targeting BAFF and APRIL, a small open label randomized controlled trial of rituximab in patients with IgAN showed no effect on proteinuria reduction, renal function, and importantly on serum levels of Gd-IgA1 and glycan-specific IgG autoantibodies, implying that peripheral B cell depletion is not an effective strategy in the management of IgAN [37]. Therefore, other B cell populations not affected by rituximab, for example plasmablasts, plasma cells, or tissue-resident B cells may play an important role in IgAN. A small open-label pilot study of the plasma cell proteasome inhibitor bortezomib demonstrated a reduction in proteinuria in eight patients with IgAN [38], and further studies of other agents are planned to examine the effects of targeting these B cell populations more specifically.

B cells express a number of TLRs, which represent an important part of the early innate immune response to invading microbial pathogens, by recognition of DAMPs (danger-associated molecular patterns) and PAMPs (pathogen-associated molecular patterns). Several TLRs, specifically -4, -9 and -10, have been implicated in the pathogenesis of IgAN [39]. Levels of TLR-4 gene expression were raised in B cells from children with IgAN and IgA vasculitis (IgAV) compared to healthy subjects [40]. Exposure to environmental antigens resulted in a higher TLR-9 gene expression and more severe renal injury in a mouse model of IgAN [41]. A small trial of hydroxychloroquine which inhibits TLR-9, and to a lesser extent TLR-7 and TLR-8, has been conducted in Chinese patients with IgAN, and was associated with a reduction in proteinuria [42]. Further larger studies in more diverse patient populations are required to see if this result can be validated. 

Spleen tyrosine kinase (Syk) has a well-established role in mediating signalling from immunoreceptors, including the B cell receptor and immunoglobulin Fc receptors, and is expressed by many cell types, including B cells, myeloid cells, and renal mesangial and tubular cells. Glomerular expression of Syk has been shown to be increased in IgAN and correlates with serum creatinine levels at the time of performing a renal biopsy [43]. In vitro, pro-inflammatory cytokine release by mesangial cells in response to IgA1 from patients with IgAN is inhibited by gene silencing of Syk, or by the Syk inhibitor fostamatinib [44]. A Phase II trial of fostamatinib in IgAN (SIGN: Syk Inhibition in IgAN) was recently completed. Interim results indicated a dose-dependent reduction in proteinuria in those with urine protein excretion >1 g/day, but this did not reach statistical significance [45]. The full results from this trial are awaited.

## 7. Complement System Inhibitors

There is mounting clinical, biochemical, and genetic evidence regarding the pivotal role of the complement cascade in the pathogenesis, disease onset, and progression of IgAN [46,47,48,49,50,51,52]. Complement (C) component C3 is co-localized with glomerular IgA in >90% of biopsies with IgAN [53]. The presence of C3 distinguishes IgAN from subclinical glomerular IgA deposition. Immunohistochemical findings of C3, C4, C4d [54], properdin, mannose binding lectin (MBL) [55], and terminal complement complex (C5b-9) deposits [56] in the mesangium of IgAN biopsy samples, and the typical absence of C1q support the involvement of the alternative and lectin pathways, rather than the classical pathway [16,57,58,59]. Evidence of complement activation in biopsies is associated with disease activity and portends a worse renal prognosis [60,61,62]. C5a is a potent local inflammatory mediator, especially via its chemoattractant and neutrophilic activating properties, and its presence in the kidney correlates with histological severity and proteinuria in IgAN [63].

Serum complement levels, such as C3 and C4, are typically normal, and in some IgAN patients, even elevated [64], as are complement components C1q and C2-C9 [64,65,66,67,68]. Nonetheless, the utility of complement proteins in the circulation as prognostic biomarkers in IgAN is still under investigation [52,61]. The elevated serum IgA:C3 ratio is typically found in IgAN patients and is a good diagnostic marker to distinguish IgAN from other glomerular diseases [69]. Studies suggest that the serum IgA:C3 ratio can also be utilized as a prognostic marker, with higher values being associated with more severe disease histology [70] and worse clinical outcomes including proteinuria, hematuria, and elevated serum creatinine levels [71]. A Korean study involving 343 patients with IgAN showed that a decreased serum C3 level (<90 mg/dL) predicted a worse outcome, which was defined by a higher rate of doubling of serum creatinine and progression to ESKD [72]. C3 is also present in IgA1-containing circulating immune complexes of patients with IgAN [73]. In a retrospective study involving 1356 Chinese IgAN patients, serum C4 levels correlated positively with proteinuria and negatively with eGFR. Furthermore, higher serum C4 levels correlated with worse tubulointerstitial injury, global sclerosis, and crescents on kidney biopsy [74]. Serum C4 levels may be an independent risk factor for IgAN progression. 

The deletion of complement factor H-related (*CFHR*) genes 1 and 3 has been identified as being protective against IgAN in genome-wide association studies [49,75,76]. Urinary excretion of complement components has also been proposed as a biomarker of the activity of IgAN. Urinary levels of Factor H and soluble C5b-9 correlated positively with proteinuria, a rise in serum creatinine levels, interstitial fibrosis, and percentage of global glomerular sclerosis, whereas urinary properdin levels were associated with only proteinuria. The urinary excretion of these biomarkers was higher in IgAN patients when compared to healthy controls [77]. Another study demonstrated increased urinary Factor H excretion in IgAN patients with more severe histologic lesions [78]. These findings have kindled a significant interest in targeting complement pathways in the management of IgAN, and ongoing clinical trials are testing several complement inhibitors using monoclonal antibodies, small molecules, and short peptides that hinder protein-complex formation and/or enzymatic reactions. However, the mechanisms linking complement activation, and subsequent levels of intact and active complement fragments, need to be further characterized to fully understand the role of the complement system in the pathogenesis, prognostic implications, and targeted therapeutics for IgAN.

Eculizumab is a humanized, recombinant, monoclonal antibody that selectively inhibits the cleavage of C5 by C5 convertase (by binding to C5 at the level of macroglobulin domain 7), thereby preventing the formation and release of the pro-inflammatory C5a and C5b components, and thereby the downstream formation of the C5b-9 membrane attack complex (MAC). Eculizumab therapy has found mixed clinical success in the management of IgAN, especially as a rescue agent in few case reports. The initial use of Eculizumab was reported from Sweden wherein a 16-year-old white male with biopsy-proven crescentic IgAN had not responded to corticosteroid and mycophenolate mofetil use, but stabilized with eculizumab treatment, although its therapeutic effects were short lived [79]. Similarly, the use of eculizumab in another 16-year-old male with crescentic IgAN resulted in transient improvement in kidney function after failure of a combination regimen consisting of corticosteroids, cyclophosphamide, and plasma exchange [80]. Eculizumab was also used as a rescue therapy in a 28-year-old male with recurrence of crescentic IgAN post kidney-transplantation but failed to salvage the allograft. It is worth mentioning that in that case, eculizumab therapy was initiated after the start of renal replacement therapy and hence it is possible that its administration was too late in the disease course [81]. Ravulizumab is a long-acting, humanized, recombinant, monoclonal antibody against C5 predicted to have similar effects to Eculizumab, that is currently being tested in a phase II clinical trial in the treatment of IgAN (ClinicalTrials.gov Identifier: NCT04564339).

C5a binds to the membrane-associated C5a receptor (C5aR) via a C-terminal C5a pentapeptide. Selectively targeting C5a offers an opportunity to dampen the local inflammation which plays a vital role in the progression of IgAN [63]. Avacopan (CCX168), a small molecule C5aR blocker, exerts its effect by binding to the C5aR surface, thereby impeding C5a binding via allosteric modulation of the C5a-binding pocket. It was evaluated in an open-label phase II trial involving seven IgAN patients (ClinicalTrials.gov Identifier: NCT02384317). At the end of 12 weeks, there was a reduction in proteinuria in six patients and UPCR decreased to <1 g/g in three patients [82]. Because avacopan does not block the downstream formation of C5b and C5b-9 MAC, as occurs with C5 inhibitors like eculizumab and ravulizumab, it has been postulated that the risk for infections with encapsulated organisms, especially belonging to the *Neisserial* species, is reduced with avacopan use. Larger studies with longer follow-up periods are warranted to confirm the efficacy of C5a inhibitors in IgAN as well as their safety.

Cemdisiran (ALN-CC5) is a synthetic, small interfering RNA (RNAi) that was designed to suppress C5 production in the liver, which can potentially limit terminal complement pathway activation and subsequent inflammation [52]. A phase II, randomized, placebo-controlled clinical trial (ClinicalTrials.gov Identifier: NCT03841448) is underway that aims to evaluate the efficacy and safety of cemdisiran in IgAN patients with persistent proteinuria >1 g/day despite optimal conservative management.

While eculizumab, ravulizumab, avacopan, and cemdisiran may be non-specific inhibitors of the distal common complement pathway, other pharmacological complement directed therapies target a specific pathway more proximally. The C3 convertase catalyzes the cleavage of C3 into C3a and C3b. This is one of the most important steps in the complement cascade and amplifies activation from the classic, alternative, and lectin pathways. The smaller, soluble C3a fragment is an anaphylatoxin that mediates inflammation. The larger subunit C3b is a highly unstable molecule and is an opsonin that covalently binds surfaces such as adjacent pathogenic cells via a reactive thioester bond, with subsequent cell phagocytosis. C3b binding can lead to C5 convertase formation by the association of C3b with C4b2a (classical and lectin complement pathways) or with C3bBb (the product of cleavage from the alternative pathway) [52,83,84]. Compstatin, a cyclic tridecapeptide; and pegcetacoplan (APL-2), a pegylated derivative of compstatin, bind to C3 and prevent its cleavage to C3a and C3b by C3 convertase [52,85]. Pegcetacoplan (APL-2) is currently being evaluated in a phase II clinical trial as a treatment option for patients with IgAN, lupus nephritis, primary membranous nephropathy or C3 glomerulopathy (ClinicalTrials.gov Identifier: NCT03453619).

The alternative complement pathway plays an important role in the essential amplification mechanism for the activation of the classical and lectin complement pathways, resulting in enhanced opsonization and generation of the terminal lytic pathway. The two proteases Factor B and Factor D play a paramount role in this tightly regulated amplification process [52,85]. Selective small-molecule reversible inhibitors of Factors B and D were developed to efficiently impede alternative complement pathway activation. Iptacopan (LNP023) is a first in class oral small molecule Factor B inhibitor. Results from a recently completed phase II clinical trial in the management of IgAN are awaited, and a phase III trial (APPLAUSE-IgAN) is currently recruiting (ClinicalTrials.gov Identifier: NCT04578834).

Mannose-binding lectin associated serine protease 2 (MASP-2) is an important component of the lectin pathway that, with MASP-1, cleaves C4 and C2 into active fragments. Thus MASP-2 triggers formation of the C3 convertase and ensuing subsequential inflammatory effects. Targeting MASP-2 inhibition can thereby curtail glomerular lectin pathway activation whilst still enabling C3 convertase to be generated via the classical and alternative pathways. Narsoplimab (OMS721) is a humanized monoclonal antibody selectively targeting MASP-2 [52,85]. In functional assays, it has shown no demonstrable effect on the classical or alternative complement pathways. In a phase II, multicenter, clinical trial, IgAN patients with proteinuria >1 g/day despite maximal tolerated RAAS blockade and baseline eGFR > 30 mL/min/1.73m^2^ were enrolled into two sub-studies based on whether they were corticosteroid-dependent or -independent at baseline. Interim analysis of both groups revealed the drug was safe, well-tolerated, and decreased proteinuria with a stable eGFR [86]. Based on this preliminary data, a randomized, double-blind, placebo-controlled, phase III clinical trial (ARTEMIS-IGAN) is underway assessing the efficacy and safety of narsoplimab in IgAN patients with persistent proteinuria >1 g/day (ClinicalTrials.gov Identifier: NCT03608033). Selvaskandan et al. reported the first case of narsoplimab use in IgA vasculitis with nephritis (IgAV-N), a condition whose pathologic features are indistinguishable from those of IgAN [87,88,89,90], presenting as a rapidly progressive glomerulonephritis with crescentic features despite the use of corticosteroids in a 21-year-old female with normal baseline serum creatinine. The patient declined cyclophosphamide and was offered narsoplimab on a compassionate use basis. The patient subsequently received 12 consecutive weekly infusions of narsoplimab (4 mg/kg) which she tolerated well without any adverse events. Her kidney function stabilized, and she successfully received a deceased-donor kidney transplant within 72 h after completing the 12th infusion of narsoplimab. There was a sustained reduction in lectin pathway activity, while classical complement pathway activity and serum IgA levels remained within the normal range and were unaffected by narsoplimab therapy. Alternate complement pathway activity was not tested in this case. Interestingly, there were no significant effects on proteinuria while on narsoplimab treatment [91].

Apart from evaluating the efficacy of various inhibitors of the complement cascade, risks associated with their usage, particularly infections, need to be carefully assessed. There is a paucity of available data regarding the safety of complement therapy inhibitors. The risk of infection depends on the level of complement pathway inhibition. C5 inhibitors primarily increase the risk of *Neisseria spp.* infections given that they block the formation of C5b and C5b-9 MAC [92]. Eculizumab use considerably elevates the risk of acquiring infections with encapsulated organisms, particularly meningococcal infections (nearly 2000-fold compared to the general population). It is therefore recommended that patients considering treatment with eculizumab or ravulizumab receive meningococcal vaccination, including the serogroups A,C,Y and W-135 conjugate vaccine and serogroup B vaccine, at least 2 weeks prior to start of therapy [93]. Alternatively, if urgent treatment is needed, patients should be treated prophylactically with ciprofloxacin until the vaccination is received [94]. C3 inhibitors are more likely to confer a more expansive infectious susceptibility necessitating vaccination against several encapsulated organisms. Nonetheless, even C3 inhibitors like compstatin do not completely dampen complement-mediated immune effects against microbes given that even reticent residual complement activity confers protection [95]. Other potential safety concerns stem from the fact that certain deficiencies of the classical complement pathway escalate the risk of developing systemic lupus erythematosus, thereby raising the potential for promoting autoimmunity with complement inhibition [95].

## 8. Non-immune Modulators

### 8.1. Endothelin Receptor Antagonists

Endothelin-1 (ET-1) exerts several physiological effects in the kidneys, including the regulation of water and sodium homeostasis. ET-1 is a growth factor for mesangial cells and has been implicated in podocyte damage, proteinuria, fibrosis, and progression of chronic kidney disease (CKD) [96]. Furthermore, urinary excretion of ET-1 correlates with the severity of kidney disease [97]. The ET-1 system is complex, consisting of a converting enzyme and two active receptors: the endothelin-A receptor (ETA-R) and endothelin-B receptor (ETB-R) [98]. The activation of the ETA-R mainly located in vascular smooth muscle cells induce extremely potent vasoconstriction, cellular proliferation, endothelial dysfunction, insulin resistance, inflammation, and fibrosis. On the other hand, ETB-Rs are mainly expressed in the vascular endothelium, and induce vasodilatation via nitric oxide and prostanoid release and aid in natriuresis [98,99]. Expression of ET-1 and ETB-R, but not the ETA-R was observed in IgAN patients with high grade proteinuria providing key evidence that activation of the endothelin system in podocytes and renal tubular cells may contribute to urinary protein loss in IgAN [100]. ET-1 expression in podocytes, and polymorphisms of the ET-1 gene have been associated with IgAN disease progression, confirmed via molecular profiling studies [101,102]. A specific ET-receptor antagonist (FR 139317) was able to suppress the development of histologic lesions and proteinuria in ddY mice with IgAN [103]. Sparsentan, a dual inhibitor of the angiotensin II type 1 (AT1) receptor and ETA-R that demonstrated significant reduction in proteinuria compared to irbesartan in patients with focal segmental glomerulosclerosis (FSGS) [104] is currently being tested in the phase III PROTECT trial (ClinicalTrials.gov Identifier: NCT03762850) evaluating its long-term renoprotective potential in IgAN. Another phase III trial (ALIGN) is evaluating the effect of Atrasentan, a selective antagonist of the ETA-R, on proteinuria reduction in IgAN patients (ClinicalTrials.gov Identifier: NCT04573478). 

### 8.2. Bardoxolone Methyl

Activation of nuclear factor erythroid 2-related factor 2 (Nrf2) and Kelch-like ECH-associated protein 1 (KEAP1) pathways by bardoxolone methyl results in the downregulation of the main proinflammatory transcription factor nuclear-factor kappa-light-chain-enhancer of activated B cells (NF-ĸB) and activation of certain antioxidative pathways [105]. In the IgAN cohort of phase II PHOENIX clinical study (ClinicalTrials.gov Identifier: NCT03366337), patients treated with bardoxolone experienced a temporary increase in eGFR of 8 mL/min/1.73m^2^ (*n* = 26, *p* < 0.0001) at week 12 compared to baseline. Historical eGFR data were available for 23 of these patients, which demonstrated that their kidney function was declining at an average annual rate of 1.2 mL/min/1.73m^2^ prior to study initiation [106]. No follow-up studies testing the benefit of bardoxolone in IgAN have been initiated so far to substantiate these preliminary results.

### 8.3. Sodium-Glucose Cotransporter 2 Inhibitors (SGLT2i)

SGLT2i are a novel class of medications recently introduced for the treatment of diabetes mellitus that exert their glucose lowering effect by inhibiting glucose entry into the proximal renal tubular cells through the SGLT2 co-transporter, thereby leading to enhanced glycosuria. A number of recent studies in patients with T2DM have demonstrated that SGLT2i wielded kidney protective effects independent of their anti-glycemic effects [107,108,109]. The DAPA-CKD study enrolled 4304 CKD patients with an eGFR of 25–75 mL/min/1.73m^2^ and albuminuria of 200–5000 mg/g who were randomized to receive the SGLT2i dapagliflozin either 10 mg/day or placebo. Participants had a mean age of 62 years with a mean baseline eGFR of 43.1 mL/min/1.73m^2^ and a median baseline albuminuria of 949 mg/g. 68% of them had T2DM. The cause of CKD was ischemic/hypertensive nephropathy in 16%, IgAN in 6% (*n* = 270; 38 with T2DM and 232 without T2DM) and FSGS in 3%. Diagnosis had been confirmed by kidney biopsy in 20% of patients. The majority (97%) were on RAAS blockade. The trial was stopped early for overwhelming efficacy. Dapagliflozin significantly reduced the risk of the primary combined endpoint of > 50% eGFR decline, onset of ESKD or renal or cardiovascular death (HR 0.61; 95% CI 0.51–0.72; *p* < 0.001). The benefit of dapagliflozin on the primary endpoint was consistent in patients with and without T2DM (HR 0.64 (95% CI 0.52–0.79) and 0.50 (95% CI 0.35–0.72), respectively; *p* for interaction = 0.024). This was also observed in patients with an eGFR < 45 or ≥ 45 mL/min/1.73m^2^ and with albuminuria ≤ 1000 or > 1000 mg/g, i.e., in patients with different stages of CKD and different severity of albuminuria. There were no statistically significant differences in DAPA-CKD in the primary endpoint between diabetics and non-diabetics, however, the hazard ratio was 22% lower for non-diabetics. Thus, the kidney benefit afforded by dapagliflozin was at least as large in patients with non-diabetic kidney disease (including ischemic/hypertensive nephropathy, IgAN and FSGS, among others) as in diabetic kidney disease. Dapagliflozin was also found to be safe in patients with CKD in this study [110].

A pre-specified analysis of the DAPA-CKD trial looking at the effects of dapagliflozin on major adverse kidney events in IgAN patients was recently published [111]. Of 270 participants with IgAN (254 (94%) confirmed by previous biopsy), 137 were randomized to dapagliflozin 10 mg/day and 133 to placebo and followed for a median duration of 2.1 years. Mean age was 51.2 years; 67.4% were male; 58.9% were Asian; 14.1% had T2DM; mean eGFR was 43.8 ± 12.2 mL/min/1.73 m^2^; and median urinary albumin-to-creatinine ratio (UACR) was 900 mg/g. Participants had similar baseline characteristics. The primary composite outcome of ≥ 50% eGFR decline, onset of ESKD or renal or cardiovascular death outcome occurred in six (4%) participants on dapagliflozin and 20 (15%) on placebo (HR 0.29; 95% CI 0.12-0.73; *p* = 0.005). The absolute risk difference was −10.7% (95% CI: −17.6, −3.7). Similar results were noted for the secondary kidney-specific outcome [HR 0.24; 95% CI 0.09–0.65; *p* = 0.002]. Five participants (4%) in the dapagliflozin group and 16 (12%) in the placebo group developed ESKD during the trial [HR 0.30; 95% CI 0.11–0.83; *p* = 0.014]. Mean rates of eGFR decline with dapagliflozin and placebo were −3.5 and −4.7 mL/min/1.73m^2^/year, respectively, resulting in a between-group difference of 1.2 mL/min/1.73m^2^ per year (95% CI −0.12, 2.51). Dapagliflozin reduced UACR by 26% relative to placebo (95% CI: −0.37, −14; *p* < 0.001). Additionally, blood pressures were lower in patients randomized to dapagliflozin. Adverse events leading to study drug discontinuation were similar with dapagliflozin and placebo, and there were fewer serious adverse events with dapagliflozin. There were no cases of diabetic ketoacidosis or major hypoglycemia in IgAN participants receiving dapagliflozin. While all patients in this trial had to be on a stable dose of a RAAS inhibitor for at least 4 weeks, it is unclear from the data whether RAAS blockade was maximized, and therefore it is difficult to know how much further improvement could have been achieved by the optimization of the currently available treatment before the addition of dapagliflozin [112]. The ongoing EMPA-KIDNEY trial (ClinicalTrials.gov Identifier: NCT03594110) has recruited a larger number of CKD patients and will likely shed more light on the safety of SGLT2i in IgAN patients. SGLT2i need to be further assessed, ideally in a dedicated trial where they are tested in addition to optimized supportive care but may be a promising addition to the management of nondiabetic glomerular diseases, including IgAN, treatment armamentarium.

## 9. Discussion

IgAN is an autoimmune disease that appears to be driven by subtle dysregulations in the adaptive and innate immune systems which we have outlined. Given its vast heterogeneity in clinical presentation and prognosis between individuals and indeed geographical locations, IgAN is unlikely to represent a single disease, but rather a common histological endpoint towards which different paths can converge. Despite being described over 50 years ago, outcomes in IgAN have remained remarkably static over the past few decades. More recently however, significant advances have been made in the understanding of the disease pathogenesis, which has driven the development of new therapeutic strategies in a number of areas that we have described in this review. It is therefore likely that new treatments will be licensed for the treatment of IgAN within the next few years. It is hoped that advances in molecular techniques will allow the capability of targeting novel therapies to an individual’s disease process at its various stages, with the ultimate aim of improving outcomes for those affected by this condition. 

## Figures and Tables

**Table 1:** Novel Therapies for Treatment of Primary IgA Nephropathy.

Agent	Mechanism of Action	Clinical Trial Design	Clinical Outcomes (Reported/Being Investigated)
***A. Targeting the Gut Mucosal Immune System***			
TRF Budesonide	Corticosteroid formulation acts on distal ileum targeting B-cells in mucosal lymphoid tissue	Randomized, double-blind, placebo-controlled Phase II clinical trial (NEFIGAN)—completed * NCT01738035	Reduction in proteinuria No change in eGFR
		Randomized, double-blind, placebo-controlled Phase III clinical trial (NefIgArd)—recruiting * NCT03643965	Effect on proteinuria Effect on eGFR
Fecal microbiota transplantation	Restoration of intestinal microecological balance	Open-Label Phase II clinical trial—recruiting * NCT03633864	Effect on proteinuria
***B. Targeting B-cells***			
Bortezomib	Semi-selective plasma cell proteasome inhibitor	Open-label Phase IV clinical trial—completed * NCT01103778	Reduction in proteinuria only in patients with T0 MEST-C score
Fostamatinib	Oral spleen tyrosine kinase inhibitor	Randomized, double-blind, placebo-controlled Phase II clinical trial—completed * NCT02112838	Non-significant reduction in proteinuria No change in eGFR
Atacicept	Blocks downstream effects of BAFF and APRIL	Randomized, double-blind, placebo-controlled Phase II clinical trial—terminated (slow enrollment) * NCT02808429	Dose-dependent reduction in proteinuria Dose-dependent reduction in immunoglobulin (particularly Gd-IgA1) levels No change in eGFR
		Randomized, double-blind, placebo-controlled Phase II clinical trial (ORIGIN)—not yet recruiting * NCT04716231	Effect on proteinuria Effect on eGFR
Blisibimod	Selective BAFF antagonist	Randomized, double-blind, placebo-controlled Phase II/III clinical trial—completed * NCT02062684	Effect on proteinuria – data analysis pending
VIS649	Monoclonal antibody against APRIL	Randomized, double-blind, placebo-controlled Phase II clinical trial (enVISion)—recruiting * NCT04287985	Effect on proteinuria Adverse events
BION-1301	Monoclonal antibody against APRIL	Open-label, non-randomized Phase II Clinical trial—*recruiting*	Adverse events Effect on proteinuria Effect on eGFR
Hydroxychloroquine	Immunomodulator, inhibits mucosal and intrarenal Toll-like receptor signaling	Randomized, double-blind, placebo-controlled Phase II clinical trial—completed * NCT02942381	Reduction in proteinuria No change in eGFR
***C. Complement System Inhibitors***			
Ravulizumab	Humanized monoclonal antibody against C5	Randomized, double blind, placebo-controlled Phase II clinical trial—recruiting * NCT04564339	Effect on proteinuria Effect on eGFR
Avacopan (CCX168)	C5a receptor blocker	Open-label Phase II clinical trial—completed * NCT02384317	Reduction in proteinuria
Cemdisiran	Small-interfering RNA inhibits synthesis of C5	Randomized, double-blind, placebo-controlled Phase II clinical trial—recruiting * NCT03841448	Effect on proteinuria
Pegcetacoplan (APL-2)	Peptide inhibitor of C3	Open-Label Phase II clinical trial—active; not recruiting * NCT03453619	Effect on proteinuria
Iptacopan (LNP023)	Oral inhibitor of complement factor B	Randomized, double blind, placebo-controlled Phase II clinical trial—active, not recruiting * NCT03373461	Effect on proteinuria
		Randomized, double blind, parallel-group, placebo-controlled Phase III clinical trial (APPLAUSE-IgAN) —recruiting * NCT04578834	Effect on proteinuria Effect on eGFR
IONIS-FB-LRx	Anti-sense inhibitor of complement factor B	Open-Label Phase II clinical trial—recruiting * NCT04014335	Effect on proteinuria
Narsoplimab (OMS721)	Monoclonal antibody against MASP-2	Open-Label Phase II clinical trial—recruiting * NCT02682407	Adverse events Effect on serum and urine complement levels Effect on proteinuria
		Randomized, double-blind, placebo-controlled Phase III clinical trial (ARTEMIS-IGAN)—recruiting * NCT03608033	Effect on proteinuria
***D. Non-Immune Modulators***			
Sparsentan	Selective antagonist of angiotensin II receptor and endothelin A receptor	Open-label Phase II clinical trial (SPARTAN)—recruiting * NCT04663204	Effect on proteinuria
		Randomized, double-blind, parallel-group, active-control Phase III clinical trial (PROTECT)—recruiting * NCT03762850	Effect on proteinuria
Atrasentan	Selective antagonist of endothelin A receptor	Open-label Phase II clinical trial (AFFINITY)—recruiting * NCT04573920	Effect on proteinuria
		Randomized, double-blind, placebo-controlled Phase III clinical trial (ALIGN)—recruiting * NCT04573478	Effect on proteinuria
Bardoxolone methyl	Semi-synthetic triterpenoid, activator of Nrf2 pathway, inhibitor of NF-ĸB pathway	Non-randomized, open-label, parallel-assignment Phase II clinical trial (PHOENIX)—completed * NCT03366337	Improvement in eGFR No serious treatment related adverse events

TRF: Targeted Release Formulation; eGFR: estimated glomerular filtration rate; BAFF: B-cell Activating Factor; APRIL: A PRoliferation-Inducing Ligand; Gd-IgA1: Galactose-deficient IgA1; RNA: Ribonucleic acid; MASP-2: Mannan-binding lectin-associated serine protease-2; Nrf2: Nuclear factor erythroid 2-related factor 2; NF-ĸB: nuclear-factor kappa-light-chain-enhancer of activated B-cells. * shows ClinicalTrials.gov Identifier. Data from www.clinicaltrials.gov (accessed on 28 April 2021).

## Data Availability

Not applicable.
